# Screening-relevant age threshold of 70 years and older is a stronger determinant for the choice of adjuvant treatment in breast cancer patients than tumor biology

**DOI:** 10.1007/s10549-017-4151-6

**Published:** 2017-02-15

**Authors:** EC Inwald, O Ortmann, M Koller, F Zeman, F Hofstädter, M Evert, G Brockhoff, M Klinkhammer-Schalke

**Affiliations:** 1 Department of Gynecology and Obstetrics, University Medical Center Regensburg, Regensburg, Germany; 2 Center for Clinical Studies, University Hospital Regensburg, Regensburg, Germany; 3 Tumor Center Regensburg, University of Regensburg, Regensburg, Germany; 4 Institute of Pathology, University of Regensburg, Regensburg, Germany

**Keywords:** Tumor biological subtypes, Breast cancer, Mammography screening, Elderly patients, Cancer registry, Overall survival

## Abstract

**Purpose:**

The 70-year threshold determines whether patients are eligible or not for the breast cancer screening program in Germany. It is not known whether this age threshold also influences the choice of adjuvant treatment and ultimate outcome.

**Methods:**

3463 patients were analyzed from the clinical cancer registry Regensburg (Germany) with primary, non-metastatic invasive breast cancer diagnosed between 2000 and 2012. The distribution of tumor biological subtypes was evaluated in breast cancer patients both in those eligible for screening (ESG, 50–69 years) and those not eligible for screening (NESG, ≥70 years). Local and systemic therapies in different subtypes as well as overall survival (OS) were analyzed.

**Results:**

2171 patients (62.7%) pertained to the ESG and 1292 patients (37.3%) referred to the NESG. The distribution of the common subtypes Luminal A, Luminal B, HER2-like, and Basal-like was comparable in both groups. Treatment varied considerably with less systemic therapies in all subtypes in patients in the NESG. Regarding local therapies, patients in the NESG also received less surgery and less radiotherapy. As to Luminal A patients, best OS was seen in patients receiving endocrine therapy (ET) (7-year OS of 95.6%) and CHT plus ET (7-year OS of 93.1%) in the ESG. In the NESG, best OS was seen in patients receiving CHT plus ET (7-year OS of 95.2%), whereas patients receiving only ET had a 7-year OS of 73.9%.

**Conclusions:**

Despite similar tumor biology, elderly patients are undertreated regarding both systemic and local therapies compared to younger patients, leading to reduced OS.

## Introduction

Breast cancer is the most common cancer in women with increasing incidence. More than 50% of breast cancer cases are diagnosed in women at the age of 60 or older [1]. However, there is a lack of evidence for specific treatment for elderly women with breast cancer [2]. Furthermore, there is a substantial underrepresentation of patients aged 65 years or older in studies about cancer treatment. This has been particularly notable in breast cancer treatment trials [3]. Indeed, elderly patients are often undertreated resulting in decreased survival [4]. In order to overcome this problem, the International Society of Geriatric Oncology (SIOG) and the European Society of Breast Cancer Specialists (EUSOMA) developed recommendations for the management of elderly patients with breast cancer [5].

Adjuvant treatment of early breast cancer is based on prognostic and predictive factors, which have been found to differ between older and young breast cancer patients. Elderly breast cancer patients more often exhibit tumors that are positive for hormone receptor (HR) expression but negative for over-expression of human epidermal growth factor receptor 2 (HER2) [6]. Moreover, it has been presumed that tumor biology in elderly patients is different from younger patients [7, 8]. Tumor biology increasingly affects treatment decisions for breast cancer patients [9]. In 2000, Perou et al. revealed that histopathological parameters correlate with the respective genetic profile [10]. In recent years, various gene expression profiling studies have enhanced our understanding of the heterogeneity and complexity of breast cancer [11, 12]. In a previous study of our group, we showed that well-established histopathological parameters, i.e., estrogen receptor (ER), progesterone receptor (PR), HER2, and Ki-67 (4-IHC) could define the four common tumor biological subtypes Luminal A, Luminal B, HER2-like, and Basal-like in routine clinical work [13]. Nevertheless, the distribution, treatment, and outcome of the tumor biological subtypes especially in elderly breast cancer patients are largely unknown.

The aim of the present study was to evaluate distribution and treatment of common tumor biological subtypes in elderly breast cancer patients based on comparison of two groups of patients with different access to medical care. Patients who are eligible for screening (50–69 years, ESG) and patients aged 70 years or older (not eligible for screening group, NESG) were compared and their local and systemic therapies in different subtypes as well as subtype-related overall survival (OS) were analyzed in a large cohort of a population-based clinical cancer registry.

## Materials and methods

### Database

In the current study, data from the Tumor Centre Regensburg (Bavaria, Germany), a high-quality population-based regional cancer registry covering a population of more than 2.2 million people of the districts of Upper Palatinate and Lower Bavaria, were analyzed. The clinical cancer registry Regensburg was founded in 1991 and currently includes the follow-up data of more than 200,000 patients. Following a stringent protocol, this cancer registry obtains a cross-sectorial documentation of all breast cancer patients in the area (*n* = 10,152 patients diagnosed between 2000 and 2012) [13]. Information about diagnosis, course of disease, therapies, and long-term follow-up are documented. Patient data originate from the University Hospital Regensburg, 53 regional hospitals, and more than 1000 practicing doctors in the region. Based on medical reports, pathology, and follow-up records, these population-based data are routinely being documented and fed into the cancer registry. Mortality data were obtained from all regional registration offices [13].

### Breast cancer screening program

Breast cancer screening by mammography is a program for the early detection of the disease. Nationwide mammography screening was a decision of the German Bundestag and Bundesrat (Lower and Upper House of the German Parliament) in 2002. In 2003, the area-wide screening program started in Bavaria and was then transferred into the German breast cancer screening 2005. Already in 2000, the first patients with mammography screening were documented in the clinical cancer registry Regensburg. The intention of the mammography screening program is to detect breast cancer early, when the tumor is still small and non-metastatic. In Germany, women between 50 and 69 years are offered the screening in form of an X-ray of the breast every two years. This screening program is the rationale of the dichotomization into patients aged 50–69 years who are eligible for screening (eligible for screening group, ESG) versus patients 70 years or older who are not eligible for screening (NESG) in the current study. The decisive factor for the classification of the two groups was the different access to medical care. Patients aged 50–69 years were eligible for mammography screening (ESG) and have controlled access in form of a structured written offer and consequently had direct access to guideline-concordant diagnosis and therapy. By contrast, patients 70 years or older lose this structured access to medical care.

### Patients´ inclusion and exclusion criteria

The present analysis included all female patients of the cancer registry with primary, non-metastatic (M0) invasive breast cancer diagnosed between 2000 and 2012 (13 years) at the age of ≥50 years. It was insignificant whether the patients participated in the mammography screening program or not. Exclusion criteria were male patients, ductal carcinoma in situ (DCIS) only, and neoadjuvant treatment. Immunohistochemical determination of 4-IHC was performed consistent with defined standards as described in detail in previous publications of our group [14–16].

### Statistical analyses

Continuous data were expressed as means ± standard deviations (SD) and categorical data as frequency counts and percentages. OS was calculated from the date of cancer diagnosis to the date of death from any cause. Living patients or patients without follow-up were classified as censored. The impact of subtypes on OS was assessed by means of a multivariable Cox regression analysis. Hazard ratios (HR) and corresponding 95% confidence intervals (CI) were calculated and considered statistically significant if CI excluded 1.0. All reported p-values were two-sided, and a *p* value of 0.05 was considered the threshold of statistical significance. Calculations were made with the software packages SPSS 22 (Chicago, EUA) and R (version 3.0.3).

## Results

### Analysis of patients´ characteristics

**Fig. 1:**
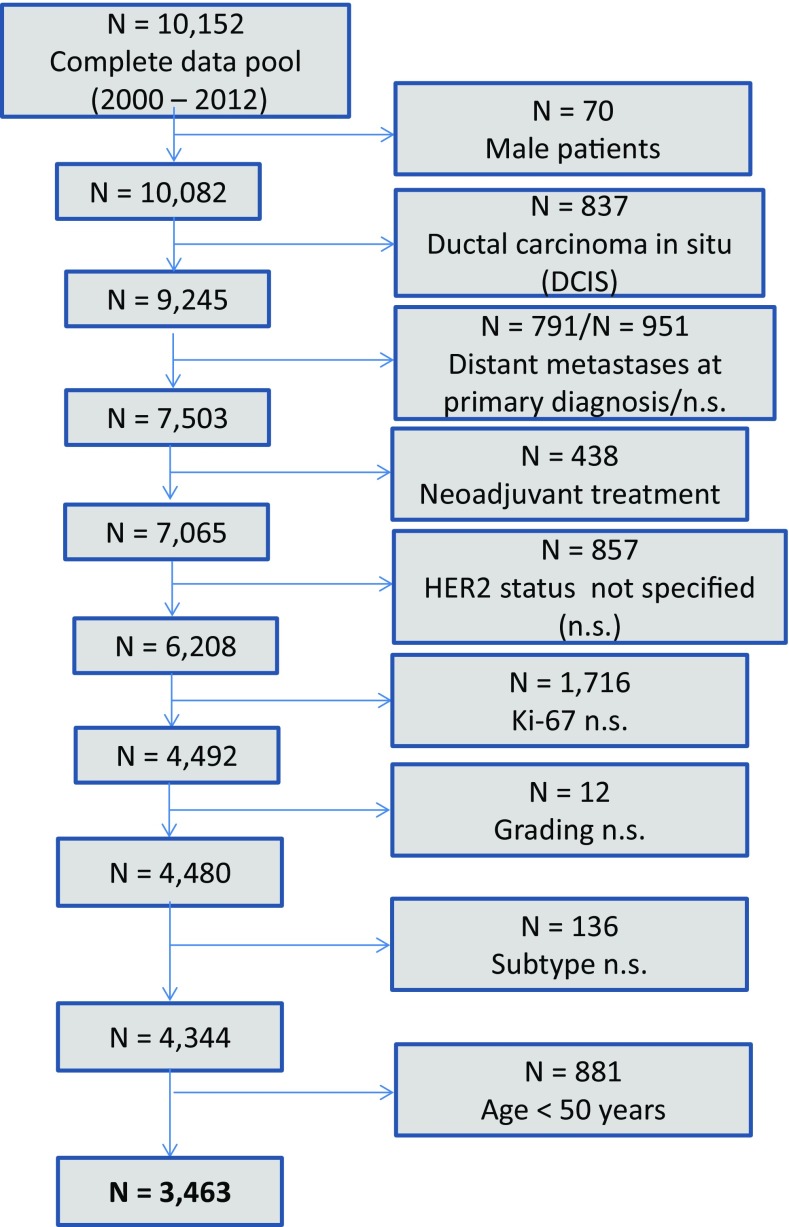
Scheme of data extraction

According to the ICD-10 classification, 4344 female patients with invasive, non-metastatic breast cancer (C50) and known ER-/PR-status, grading, HER2, Ki-67, and subtype were extracted from the total pool of breast cancer patients (Fig. 1). 881 of these patients were at the age of <50 years and accordingly excluded. Thus, a total of 3463 breast cancer patients were included in the following analyses. 2171 patients (62.7%) pertained to the ESG at the age of 50–69 years (mean ± SD: 60 ± 6). 1292 patients (37.3%) referred to the NESG aged 70 years or older (mean ± SD: 77 ± 5). Additionally, parameters of tumor biological subtypes were analyzed (Table 1). Regarding receptor status, HER2, and Ki-67, distributions were comparable with the ESG and the NESG. The most common type of grading for both the ESG and the NESG was G2. However, in the ESG, more G1 tumors were found (20.6%) than in the NESG (14.6%) (Table 1).

**Table 1:** Parameters for subtypes and classification of subtypes compared between patients aged 50–69 years (ESG) and patients ≥70 years (NESG)

Parameter	ESG (*n* = 2171/62.7%)	NESG (*n* = 1292/37.3%)	Total (*n* = 3463/100%)
Age (year), mean ± SD)	59.7 ± 6	77.1 ± 5	66.2 ± 10
Estrogen receptor, *n* (%)			
Positive	1873 (86.3)	1132 (87.6)	3005 (86.8)
Negative	298 (13.7)	160 (12.4)	458 (13.2)
Progesterone receptor, *n* (%)			
Positive	1675 (77.2)	1000 (77.4)	2675 (77.2)
Negative	496 (22.8)	292 (22.6)	788 (22.8)
Receptor Status, *n* (%)			
ER + PR+	1651 (76.0)	984 (76.2)	2635 (76.1)
ER + PR−	222 (10.2)	148 (11.5)	370 (10.7)
ER−PR+	24 (1.1)	16 (1.2)	40 (1.2)
ER−PR−	274 (12.6)	144 (11.1)	418 (12.1)
Grading, *n* (%)			
G1	448 (20.6)	188 (14.6)	636 (18.4)
G2	1273 (58.6)	820 (63.5)	2093 (60.4)
G3	450 (20.7)	284 (22.0)	734 (21.2)
HER2 Status, *n* (%)			
Positive	376 (17.3)	208 (16.1)	584 (16.9)
Negative	1795 (82.7)	1084 (83.9)	2879 (83.1)
Ki-67 categories [%], *n* (%)			
0–15	1276 (58.8)	761 (58.9)	2037 (58.8)
16–25	401 (18.5)	258 (20.0)	659 (19.0)
26–35	204 (9.4)	125 (9.7)	329 (9.5)
36–45	104 (4.8)	57 (4.4)	161 (4.6)
>45	186 (8.6)	91 (7.0)	277 (8.0)
Classification of subtypes, *n* (%)			
Luminal A	1111 (51.2)	659 (51.0)	1770 (51.1)
Luminal B	504 (23.2)	333 (25.8)	837 (24.2)
HER2-like	376 (17.3)	208 (16.1)	584 (16.9)
Basal-like	180 (8.3)	92 (7.1)	272 (7.9)

### Classification of tumor biological subtypes

Selection criteria for classification of subtypes are shown in the appendix (Table 6) according to the 2011 St Gallen Consensus Conference [17] and a modification of the original classification by Perou et al. [10] as described in a previous study of our group [13]. The most common subtype was Luminal A (*n* = 1770/51.1%) both in the ESG (*n* = 1111/51.2%) and in the NESG (*n* = 659/51.0%). Luminal B was the second most frequent entity in the ESG (*n* = 504/23.2%) as well as in the NESG (*n* = 333/25.8%). Few patients had the triple-negative Basal-like subtype (*n* = 180/8.3% vs. *n* = 92/7.1%) (Table 1).

### Systemic therapies based on age (ESG versus NESG) and subtype

Systemic therapies varied according to age (Table 2). The most common type of treatment was endocrine therapy (ET) both in the ESG (*n* = 974/44.9%) and in the NESG (*n* = 745/57.7%). Patients in the ESG received chemotherapy (CHT) plus ET (*n* = 614/28.3%) more often than patients in the NESG (*n* = 89/6.9%). Remarkably, 15.8% of all patients received no adjuvant therapy at all or other non-guideline adherent treatment (8.5% of patients in the ESG vs. 27.9% of patients in the NSG). Moreover, systemic therapies based on subtype were analyzed. Luminal A patients predominantly received only ET (*n* = 700/63.0% in the ESG vs. *n* = 455/69.0% in the NESG) followed by CHT plus ET in the ESG (*n* = 296/26.6% vs. *n* = 42/6.4% in the NESG) (Table 2). Regarding Luminal B, patients in the ESG mostly obtained CHT plus ET (*n* = 240/47.6%), whereas patients in the NESG mainly received only ET (*n* = 212/63.7%). Patients with HER2-like subtype hardly received guideline-concordant therapy with trastuzumab. Only 45.6% of patients in the ESG and 21.6% of patients in the NESG were given trastuzumab ± CHT and ± ET. More than one third of HER2-like patients in the NESG (*n* = 70/33.7%) received no adjuvant therapy. With respect to Basal-like subtype, the most common type of adjuvant therapy in the ESG was CHT (*n* = 140/77.8%), whereas 60.9% of patients in the NESG (*n* = 56) received no adjuvant therapy at all. Only 35.9% (*n* = 33) of patients with Basal-like subtype in the NESG received CHT (Table 2).

**Table 2:** Systemic therapies based on subtype in patients aged 50–69 years (ESG) and patients ≥70 years (NESG), *n* = 3463 patients

	Luminal A		Luminal B		HER2-like		Basal-like		Total	
	ESG(%)	NESG (%)	ESG (%)	NESG (%)	ESG (%)	NESG (%)	ESG(%)	NESG (%)	ESG (%)	NESG (%)
ET (*n* = 1719/49.6%)	700/63.0	455/69.0	205/40.7	212/63.7	68/18.1	76/36.5	1/0.6	2/2.2	974/44.9	745/57.7
CHT + ET (*n* = 703/20.3%)	296/26.6	42/6.4	240/47.6	39/11.7	70/18.6	7/3.4	8/4.4	1/1.1	614/28.3	89/6.9
CHT + ET + Trastuzumab (*n* = 128/3.7%)	–	–	–	–	104/27.7	24/11.5	–	–	104/4.8	24/1.9
CHT + Trastuzumab (*n* = 75/2.2%)	–	–	–	–	60/16.0	15/7.2	–	–	60/2.8	15/1.2
ET + Trastuzumab (*n* = 13/0.4%)	–	–	–	–	7/1.9	6/2.9	–	–	7/0.3	6/0.5
CHT (*n* = 279/8.1%)	25/2.3	7/1.1	26/5.2	1/0.3	37/9.8	10/4.8	140/77.8	33/35.9	228/10.5	51/3.9
None (*n* = 546/15.8%)	90/8.1	155/23.5	33/6.5%	81/24.3	30/7.9	70/33.7	31/17.2	56/60.9	184/8.5	362/27.9
Total	1111/51.2	659/51.0	504/23.2	333/25.8	376/17.3	208/16.1	180/8.3	92/7.1	2171/62.7	1292/37.3

To elucidate reasons for the insufficient realization of different therapies, we further analyzed the patients with respect to their concomitant diseases. In total, 1014 patients (29.3%) had at least one serious concomitant disease. 123 patients (3.6%) had no co-morbidity, and in 2326 patients (67.2%), concomitant diseases were not documented. The majority of patients (63.2%) suffered from cardiopulmonary disease. Others had metabolic (10.3%), mental (6.9%), gastrointestinal/hepatic/renal disorders (4.1%) or disorders of different cast (15.5%).

### Analysis of local therapies

In addition to systemic therapies, local therapies, i.e., surgery and radiotherapy, were analyzed. Most of the patients received primary surgery in the ESG (*n* = 2160/99.5%) as well as in the NESG (*n* = 1247/96.5%) (Table 3). Breast conserving therapy (BCT) was conducted significantly more often in the ESG than in the NESG (78.9 vs. 52.9%). Likewise, more patients in the ESG received guideline-concordant radiotherapy post BCT (92.8 vs. 80.4%).

**Table 3:** Local therapies: primary surgery and whole-breast radiotherapy (WBRT) compared between patients aged 50–69 years (ESG) and patients ≥70 years (NESG)

	ESG (*n* = 2171) (%)	NESG (*n* = 1292) (%)	Total (*n* = 3463) (%)
Primary surgery			
Yes	2160 (99.5)	1247 (96.5)	3407 (98.4)
No	11 (0.5)	45 (3.5)	56 (1.6)
Type of surgery			
Breast conserving (BCT)	1714 (78.9)	684 (52.9)	2398 (69.2)
Mastectomy	429 (19.8)	541 (41.9)	970 (28.0)
Unknown	28 (1.3)	67 (5.2)	95 (2.7)
WBRT post BCT			
Yes	1590 (92.8)	550 (80.4)	2140 (89.2)
No	124 (7.2)	134 (19.6)	258 (10.8)
WBRT post mastectomy			
Yes	201 (46.9)	142 (26.2)	343 (35.4)
No	228 (53.1)	399 (73.8)	627 (64.6)

### Survival analyses within different subtypes

**Fig. 2:**
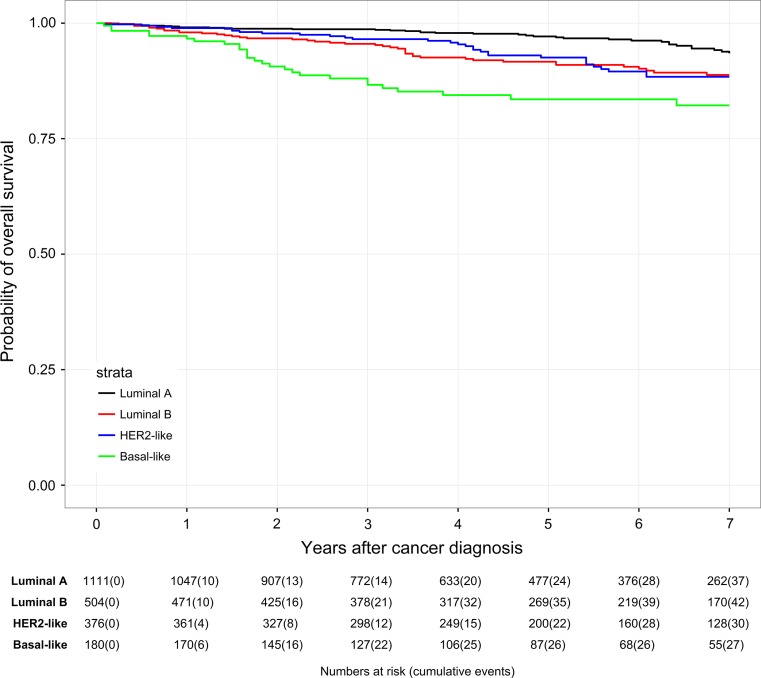
Kaplan–Meier plot of overall survival in years of patients aged 50–69 years (ESG) based on subtypes

**Fig. 3:**
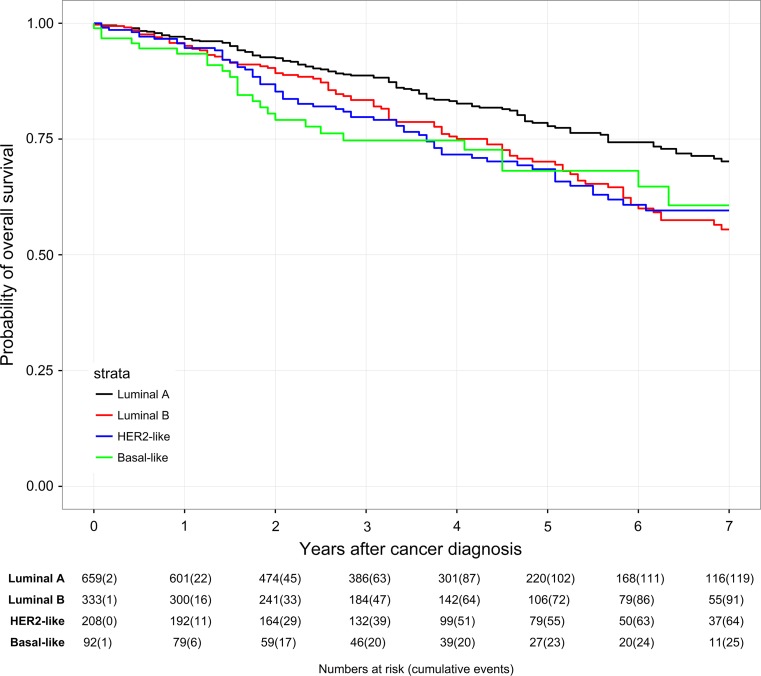
Kaplan–Meier plot of overall survival in years of patients aged ≥70 years (NESG) based on subtypes

Patients in the ESG generally had better survival rates than in the NESG (Table 4; Figs. 2, 3). Best OS was found in Luminal A tumors both in the ESG and in the NESG (7-year OS rate of 93.8 vs. 70.2%). OS rates of Luminal B tumors and HER2-like tumors were comparable in the ESG (7-year OS rate of 88.8 vs. 88.4%). In the NESG, OS of HER2-like patients (7-year OS rate of 59.6%) was comparable with Basal-like patients (7-year OS rate of 60.7%). In the ESG, the lowest OS was found in the Basal-like subtype (7-year OS rate of 82.2%). In the NESG, the lowest OS was found in the Luminal B subtype (7-year OS of 55.5%).

**Table 4:** Overall survival of patients within different subtypes compared between patients aged 50–69 years (ESG) and patients ≥70 years (NESG)

	3-y-OS (%)	5-y-OS (%)	7-y-OS (%)
ESG			
Luminal A N = 1111 → 52 events	98.7	97.1	93.8
Luminal B N = 504 → 51 events	95.5	91.6	88.8
HER2-like N = 376 → 34 events	96.5	92.6	88.4
Basal-like N = 180 → 27 events	88.0	83.5	82.2
NESG			
Luminal A N = 659 → 128 events	88.7	78.5	70.2
Luminal B N = 333 → 105 events	83.4	70.1	55.5
HER2-like N = 208 → 68 events	79.7	68.5	59.6
Basal-like N = 92 → 27 events	74.7	68.1	60.7

### Survival analyses based on subtypes and systemic therapies

 Depending on various systemic therapies, OS rates within the different subtypes and age groups differed remarkably (See appendix Tables 7 and 8). As to Luminal A patients, best OS was seen in patients receiving ET (7-year OS of 95.6%) and CHT plus ET (7-year OS of 93.1%) in the ESG (See appendix Table 7). In the NESG, best OS was seen in patients treated with CHT plus ET (7-year OS of 95.2%), whereas patients with only ET treatment had a 7-year OS of 73.9% (See appendix Table 8). Concerning Luminal B, again best OS was seen in patients receiving ET (7-year OS of 92.1%) and CHT plus ET (7-year OS of 88.2%) in the ESG (See appendix Table 7). In the NESG, best OS was seen in patients receiving CHT plus ET (7-year OS of 71.0%). By depriving patients in the NESG from adjuvant therapy, their 7-year OS was reduced to 36.3% (See appendix Table 8). In the HER2-like subtype, the effect of adjuvant trastuzumab was clearly seen both in the ESG and NESG. Patients in the ESG receiving CHT plus trastuzumab had a 7-year OS of 93.9% compared to those patients receiving CHT plus ET plus trastuzumab with a 7-year OS of 92.9%. Patients in the NESG treated with CHT plus ET plus trastuzumab had a 7-year OS of 82.8%. HER2-like patients receiving only CHT had comparatively worse outcome both in the ESG (7-year OS of 75.4%) and in the NESG (7-year OS of 50.0%). Referring to Basal-like subtype application of CHT led to improved survival rates in both ESG (7-year OS of 85.5%) and NESG (7-year OS of 77.0%).

A Cox regression model (Table 5) provided further evidence that the best OS was seen in Luminal A patients. The lowest OS was seen in patients with Basal-like tumors both in the ESG and in the NESG (HR = 2.27, 95% CI 1.29–3.98, *P* = 0.004 vs. HR = 1.68, 95% CI 1.01–2.79, *P* = 0.045). Kaplan–Meier plots of OS in years based on subtypes in the ESG and in the NESG are shown in Figs. 2 and 3.

**Table 5:** Multivariable Cox proportional hazard model on overall survival

	ESG (*n* = 2155)			NESG (*n* = 1233)			Total (*n* = 3388)		
	HR	95% CI	*P* value	HR	95% CI	*P* value	HR	95% CI	*P* value
Subtypes									
Luminal A	1			1			1		
Luminal B	1.35	0.88, 2.06	0.171	1.57	1.15, 2.14	**0.005**	1.51	1.17, 1.92	**0.001**
HER2-like	1.07	0.66, 1.75	0.778	1.56	1.11, 2.21	**0.012**	1.41	1.06, 1.87	**0.018**
Basal-like	2.27	1.29, 3.98	**0.004**	1.68	1.01, 2.79	**0.045**	1.93	1.33, 2.79	**0.001**

Statistically significant results are shown in bold type

Multivariable models are adjusted for age, tumor size, nodal status, grading, and histology

## Discussion

Decisions on treatment of breast cancer patients are based on national [18] and international guidelines [19]. However, these recommendations do not consider age-specific characteristics. There is a lack of evidence on the optimal management of elderly patients [3]. Thus, due to increasing life expectancy, the treatment of elderly patients is an emerging clinical problem [20].

A main cause for non-adherence to guideline recommendations may be the existence of co-morbidities of which elderly patients are more often affected than younger ones. Co-morbidities, especially cardiovascular diseases, may also be the cause of reduction of OS.

In the present study, we investigated the distribution of tumor biological subtypes in elderly patients both in the ESG (50–69 years) and the NESG (≥70 years) of breast cancer patients. Further, we studied local and systemic therapies in different subtypes as well as subtype-related OS by analyzing data of a large cohort of a clinical cancer registry. The distribution of the four common subtypes Luminal A, Luminal B, HER2-like, and Basal-like was quite comparable in the ESG versus the NESG. Luminal A tumors were found as often in the ESG (51.2%) as in the NESG (51.0%), whereas a slight increase of Luminal B tumors (25.8 vs. 23.2%) and a declining tendency of HER2-like (16.1 vs. 17.3%) and Basal-like tumors (7.1 vs. 8.3%) in the NESG was detected (Table 1). These results are in line with a study by Jenkins et al. who characterized the incidence of breast cancer patients by molecular subtypes and age using the PAM50 algorithm [21]. In this study, the incidence of Luminal A and B tumors increased with age (*P* < 0.01, *P* < 0.001), whereas the percentage of basal-like tumors decreased (*P* < 0.001) [21].

Systemic therapies varied according to age. Patients in the ESG received CHT ± ET more often than patients in the NESG (Table 2). However, according to the SIOG guidelines, there is no evidence to support differential use of specific CHT or dose reductions in older patients compared with younger ones [5]. As described in our study and in line with Cappellani et al., breast cancer in the elderly is not less aggressive compared to younger patients [22]. In particular, prognostic and predictive factors are identical [22]. A meta-analysis of the Early Breast Cancer Trialists´ Collaborative group (EBCTCG) with 15 years of follow-up on more than 100,000 women enrolled in breast cancer clinical trials evaluated adjuvant ET and CHT in detail [23]. They documented statistically significant benefits of adjuvant CHT to reduce breast cancer recurrence and mortality in women aged 50–69 years [23].

A retrospective study by the Cancer and Leukemia Group B (CALGB) noticed that older and younger women had similar reductions in breast cancer mortality from regimens containing more CHT [24]. Likewise, Muss et al. demonstrated that in women aged 65 years or older, standard adjuvant poly-chemotherapy is superior to a single-agent CHT (capecitabine) in patients with early-stage breast cancer [25].

Especially in patients with Luminal B tumors, the missing ET ± CHT led to worse outcomes both in the ESG and in the NESG (See appendix Tables 7 and 8). In line with results from Kruiff et al., this might be explained by the fact that these tumors may benefit more from CHT than other subtypes due to their high proliferative characteristics [6]. However, the problem of identifying older patients who will benefit from adjuvant CHT and to weigh potential survival advantages versus serious side effects has not been solved [26].

In contrast to CHT, patients in the NESG received more often ET only (Table 2) with 69.0% (*n* = 455) of Luminal A patients compared to 63.0% (*n* = 700) in the ESG. With respect to Luminal B patients, these differences were even more distinct. 40.7% (*n* = 205) of patients in the ESG received ET in comparison with 63.7% (*n* = 212) of patients in the NESG. Likewise, a study analyzing data from the Netherlands Cancer Registry demonstrated that the percentage of patients who received ET only increased with age for all stages [27].

For HER2-like positive patients, the application of trastuzumab in combination with CHT represents the gold standard in the adjuvant setting [28, 29]. Also, in elderly patients with HER2-positive breast cancer the use of trastuzumab should be considered as standard of care [30], even though careful management regarding mainly cardiovascular side effects is essential [31, 32]. A subgroup analysis from the herceptin adjuvant study (HERA) showed an effect of trastuzumab independent of age [33]. In line with the findings of Grumpelt et al., we observed that the use of trastuzumab was insufficient both in the ESG and the NESG [34].

Withholding basal-like patients, CHT resulted in exceeding low OS rates in both subgroups. Patients with basal-like breast cancer in the ESG receiving CHT had a 7-year OS rate of 85.5% in contrast to those patients receiving no adjuvant therapy with a 7-year OS rate of 66.9% (See appendix Table 7). Analogous to this, 7-year OS rate in the NESG deteriorated to 48.5 versus 77.0% in patients with Basal-like tumors without CHT (See appendix Table 8). Two retrospective studies of the Surveillance, Epidemiology and End Results (SEER) database revealed that adjuvant CHT improves OS in geriatric patients aged older than 65 years with ER-negative tumors [35, 36]. In an observational study of 1711 women aged ≥66 years with ER-negative breast cancer, multivariate regression analysis showed that CHT led to a 15% reduction in risk of death from any cause, in comparison with patients without CHT (HR = 0.83, 95% CI 0.74–0.92) [35]. Our results are consistent with these findings.

With respect to local therapies, breast conserving therapy (BCT) is the standard of care for operable breast cancer plus whole-breast radiotherapy (WBRT) [19]. Nevertheless, patients in the NESG received less surgery and less WBRT than patients in the ESG. This observation corresponds with a Dutch population-based study selecting 2336 female breast cancer patients ≥60 years versus ≥80 years between 2001 and 2006. The proportion of patients undergoing surgery decreased with increasing age: 99% for patients aged 60–69 years, 98% for patients aged 70–79 years, and 83% for patients ≥80 years old [37]. Patients in the ESG mostly received BCT (78.9%), whereas patients in the NESG received BCT only in 52.9%, but mastectomy in 41.9% (Table 3). A study by Rocco et al. [38] who analyzed treatment and outcomes of 449 women aged ≥65 years compared to 1049 younger patients showed higher rates of mastectomy in older patients. 72% (*n* = 324) of patients older than 65 years got mastectomy compared to 28% (*n* = 125) with BCT [38].

Omission of WBRT after BCT in elderly breast cancer patients remains a controversial issue, particularly because most randomized trials analyzing WBRT excluded patients older than 70 years. Radiotherapy after primary surgery was performed less frequently in the oldest age group in a study by Weggelaar et al. [37] agreeing with our results and with previous studies reporting less loco-regional surgery and frequent omission of radiotherapy in elderly patients [34, 36, 39–41].

## Conclusion

In conclusion, by means of analyzing data from a large cohort of a regional population-based clinical cancer registry, we demonstrated that elderly patients (≥70 years) are considerably undertreated as compared to younger patients (50–69 years) regarding both systemic and local therapies. Biology of tumors diagnosed in elderly and younger patients did not differ. Not surprisingly, OS is generally lower in elderly patients than in younger patients. However, if elderly patients receive adjuvant therapies according to current guidelines, their cancer-related OS is not lower than in younger patients.

Balancing the potential benefits and risks of different treatment methods in elderly patients remains challenging. Future studies should target to create specific geriatric screening methods for elderly breast cancer patients that can facilitate the selection of optimal treatment.

## Tables

See Tables 6, 7, and 8

**Table 6:** Classification of subtypes (*n* = 3463 patients)

Luminal A (*n* = 1770/51.1%)	Luminal B (*n* = 837/24.2%)	HER2-like (*n* = 584/16.9%)	Basal-like (*n* = 272/7.9%)
ER+ PR+	ER+ PR+	ER+ PR+	ER− PR−
ER+ PR−	ER+ PR−	ER+ PR−	
ER−PR+	ER−PR+	ER− PR+	
		ER− PR−	
G1	G1	G1	G1
G2	G2	G2	G2
	G3	G3	G3
HER2−	HER2−	HER2+	HER2−
Ki-67 ≤15%	Ki-67: >15%	Any Ki-67	Any Ki-67

**Table 7:** Overall survival based on subtype and systemic therapies in patients aged 50-69 years (ESG)

ESG	3-year OS (%)	5-year OS (%)	6-year OS (%)	7-year OS (%)
Luminal A (*n* = 1111)				
ET (*n* = 700 → 17 events)	99.4	98.4	98.0	95.6
CHT + ET (*n* = 296 → 24 events)	99.0	96.4	94.9	93.1
CHT (*n* = 25 → 2 events)	92.0	92.0	92.0	92.0
Other (*n* = 90 → 9 events)	92.2	89.4	85.4	78.8
Luminal B (*n* = 504)				
ET (*n* = 205 → 17 events)	97.5	94.1	94.1	92.1
CHT + ET (*n* = 240 → 23 events)	97.3	92.2	90.0	88.2
CHT (*n* = 26 → 5 events)	81.7	81.7	81.7	81.7
Other (*n* = 33 → 6 events)	74.2	74.2	74.2	74.2
HER2-like (*n* = 376)				
ET + Trastuzumab (*n* = 7 → 0 event)	–	–	–	–
ET (*n* = 68 → 6 events)	98.5	93.2	88.8	88.8
CHT + ET (*n* = 70 → 9 events)	100	95.0	89.7	87.8
CHT + ET + Trastuzumab (*n* = 104 → 4 events)	97.6	96.0	96.0	92.2
CHT + Trastuzumab (*n* = 60 → 2 events)	98.2	98.2	93.9	93.9
CHT (*n* = 37 → 10 events)	82.3	75.4	75.4	75.4
Other (*n* = 30 → 3 events)	93.1	86.9	86.9	86.9
Basal-like (*n* = 180)				
ET (*n* = 1 → 0 event)	–	–	–	–
CHT + ET (*n* = 8 → 1 event)	87.5	87.5	87.5	87.5
CHT (*n* = 140 → 16 events)	92.3	87.3	87.3	85.5
Other (*n* = 31 → 10 events)	66.9	66.9	66.9	66.9

**Table 8:** Overall survival based on subtype and systemic therapies in patients ≥70 years (NESG)

NESG	3-year OS (%)	5-year OS (%)	6-year OS (%)	7-year OS (%)
Luminal A (*n* = 659)				
ET (*n* = 455 → 77 events)	92.0	80.8	77.3	73.9
CHT + ET (*n* = 42 → 3 events)	95.2	95.2	95.2	95.2
CHT (*n* = 7 → 3 events)	85.7	64.3	42.9	42.9
Other (*n* = 155 → 45 events)	75.6	66.3	59.3	49.1
Luminal B (*n* = 333)				
ET (*n* = 212 → 61 events)	87.7	74.1	64.6	58.8
CHT + ET (*n* = 39 → 9 events)	91.5	83.9	71.0	71.0
CHT (*n* = 1 → 0 event)	–	–	–	–
Other (*n* = 81 → 35 events)	66.4	50.6	43.6	36.3
HER2-like (*n* = 208)				
ET + Trastuzumab (*n* = 6 → 0 event)	–	–	–	–
ET (*n* = 76 → 20 events)	87.3	81.5	72.0	72.0
CHT + ET (*n* = 7 → 2 events)	85.7	85.9	71.4	71.4
CHT + ET + Trastuzumab (*n* = 24 → 3 events)	91.3	82.2	82.2	82.2
CHT + Trastuzumab (*n* = 15 → 3 events)	92.9	82.5	68.8	68.8
CHT (*n* = 10 → 5 events)	70.0	50.0	50.0	50.0
Other (*n* = 70 → 35 events)	61.8	41.6	33.9	29.6
Basal-like (*n* = 92)				
ET (*n* = 2 → 0 event)	–	–	–	–
CHT + ET (*n* = 1 → 0 event)	–	–	–	–
CHT (*n* = 33 → 6 events)	88.8	77.0	77.0	77.0
Other (*n* = 56 → 21 events)	64.6	61.0	61.0	48.5
